# The Relations Among Anxiety, Movie‐Watching, and in‐Scanner Motion

**DOI:** 10.1002/hbm.70163

**Published:** 2025-03-05

**Authors:** Peter A. Kirk, Purnima Qamar, Andre Zugman, Rany Abend, Samuel Frank, Grace V. Ringlein, Laura Jett, Gwyneth A. L. DeLap, Anita Harrewijn, Daniel S. Pine, Katharina Kircanski

**Affiliations:** ^1^ Emotion and Development Branch National Institute of Mental Health Bethesda Maryland USA; ^2^ School of Psychology Reichman University Herzliya Israel; ^3^ Department of Biostatistics, Bloomberg School of Public Health Johns Hopkins University Baltimore Maryland USA; ^4^ Department of Psychology University of Wisconsin‐Madison Madison Wisconsin USA; ^5^ Department of Psychology University of Rochester Rochester New York USA; ^6^ Department of Psychology, Education and Child Studies Erasmus University Rotterdam Rotterdam the Netherlands

## Abstract

Movie‐watching fMRI has emerged as a theoretically viable platform for studying neurobiological substrates of affective states and emotional disorders such as pathological anxiety. However, using anxiety‐inducing movie clips to probe relevant states impacted by psychopathology could risk exacerbating in‐scanner movement, decreasing signal quality/quantity and thus statistical power. This could be especially problematic in target populations such as children who typically move more in the scanner. Consequently, we assessed: (1) the extent to which an anxiety‐inducing movie clip altered in‐scanner data quality (movement, censoring, and DVARS) in a pediatric sample with and without anxiety disorders (*n* = 78); and (2) investigated interactions between anxiety symptoms and movie‐attenuated motion in a highly powered, transdiagnostic pediatric sample (*n* = 2058). Our results suggest anxiogenic movie‐watching in fact reduces in‐scanner movement compared to resting‐state, increasing the quantity/quality of data. In one measure, pathological anxiety appeared to impact movie‐attenuated motion, but the effect was small. Given potential boosts to data quality, future developmental neuroimaging studies of anxiety may benefit from the use of movie paradigms.

## Graphical abstract

Children and adolescents are a difficult‐to‐image population due their tendency to move during scanning sessions. Our results suggest youth show lower in‐scanner movement when viewing anxiogenic and non‐anxiogenic movie clips compared to rest.

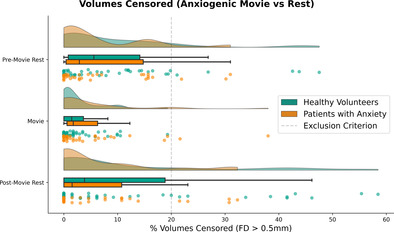

## 1 Introduction

Pathological anxiety typically emerges during childhood and adolescence, conferring high risk for multiple negative outcomes (Beesdo et al. 2009). Delineating pathophysiological substrates in anxious youth is crucial for efforts to improve early diagnosis and intervention. While neuroimaging research in anxiety traditionally uses tightly controlled experimental designs, recent work has begun to include naturalistic stimuli through movie‐watching functional magnetic resonance imaging (fMRI). Movies provide contextually rich, dynamic, and ecologically engaging stimuli offering a unique opportunity to probe multiple affective dimensions related to psychopathology, including anxiety, with fMRI (Kinreich et al. 2011; Kirk et al. 2022a, 2022b, 2023). However, high in‐scanner motion, commonly reported with pediatric samples (Satterthwaite et al. 2012; Van Dijk et al. 2012), is one of the largest detriments to signal quality (Friston et al. 1996) and may thus potentially negatively affect movie‐watching fMRI research in anxious youth. In this paper, we ask: how do anxiety and movie‐watching impact movement during fMRI in children and adolescents?

Clinical neuroscience investigates the pathophysiology driving alterations in emotional and cognitive states, typically via computerized lab tasks. While these have provided many contributions to the field, many of the paradigms suffer from low ecological validity, moderate reliability, and participant boredom, including among youth (Haller et al. 2022; Jangraw et al. 2023; Vigliocco et al. 2024). Movie‐watching may complement traditional task‐based research designs. Movies can probe diverse facets of cognition and emotions in a more engaging, naturalistic manner and are able to elicit reliable engagement of functional networks (Finn and Bandettini 2021; Kirk et al. 2022a, 2022b; Morgenroth et al. 2024; Vanderwal et al. 2017). Moreover, watching emotionally neutral and pleasant movies appears useful for reducing in‐scanner motion amongst children and adolescents, thus boosting signal quality and statistical power (Frew et al. 2022; Van derwal et al. 2015; Vanderwal et al. 2019). However, many studies of psychopathology require the elicitation of negative affective states, which neutral and pleasant movies do not typically evoke. Anxiety researchers specifically probe pathology‐relevant processes by inducing states of potential threat and anxiety in the scanner.

Induced anxiety might relate to motion artifacts in the MRI environment (Dantendorfer et al. 1997; Walworth 2010) and reduced signal quality. It is plausible that inducing states of anxiety through movies may fail to confer the same benefits as neutral and pleasant movies. Worse, they could increase in‐scanner motion, confounding studies on the neural substrates of anxiety. Moreover, the general benefits of movies attenuating motion might not be apparent in individuals with high levels of anxiety symptoms. It is critical to evaluate these possibilities in individuals with anxiety, and particularly among youth for whom motion may be high due to their younger age (Satterthwaite et al. 2012). To our knowledge, despite the increased use of movies in fMRI research (Sonkusare et al. 2019; Vanderwal et al. 2019), no study to date has explicitly investigated the relationship between anxiety and in‐scanner movement in children and adolescents during movie‐watching. Data acquired from treatment‐seeking patients may be also most generalizable to clinical intervention studies designed to target neural circuits engaged by threat and anxiety. In this study, we therefore sought to characterize in‐scanner movement and signal quality during movie‐watching (anxiogenic and non‐anxiogenic) in pediatric samples as a function of anxiety symptoms.

## 2 Methods

### 2.1 Datasets

The present study made use of two datasets, described below (summarized in Table 1), to assess in‐scanner motion and data quality. The first, primary analysis was based on an NIH dataset of children and adolescents with and without and anxiety disorder, which constitutes our main results. The Healthy Brain Network dataset (Alexander et al. 2017) was used as a secondary, supplemental analysis to explore interactions with transdiagnostic anxiety symptoms, reported at the end of *results*. Code and data for those who consented to share are publicly available on Open Science Foundation (https://osf.io/6v42r/).

**TABLE 1:** Summary of data conditions and participants.

Dataset	NIH		Healthy brain network
Conditions	Anxiogenic movie rest		Non‐anxiogenic movies rest
Diagnosis	Healthy volunteers	Patients with anxiety	Transdiagnostic
Sample	44	34	2058
Age			
Range	8.1–18.0	9.6–17.9	5.0–21.9
Mean	14.2	14.4	11.0
SD	2.5	1.9	3.2
Sex			
Female	27	24	738
Male	17	10	1320

#### 2.1.1 NIH Sample

Following exclusion of 6 subjects due to aborted scans or failed preprocessing, the final NIH sample included 78 participants recruited in person at the National Institute of Mental Health in Bethesda, MD (Table 1). Of those, 44 were healthy volunteers, and 34 were treatment‐seeking volunteers with an anxiety disorder (as established with the K‐SADS‐PL interview; Kaufman et al. 1997). Age range criteria for this dataset was based on two factors: (1) selection parameters for research on treatment of child and adolescent anxiety (Walkup et al. 2001); and (2) participants below 8 were not recruited due to considerations regarding tolerance for the anxiogenic stimulus. A Brown‐Forsythe test did not suggest a significant difference in the variance of age between diagnostic groups (*p* = 0.091; for age distributions, see Supplement 1). Participants underwent whole‐brain echo planar imaging on one of two GE MR750 3 T MRIs with the following parameters: 3.8 mm^3^ voxels, TR = 2000 ms, TE = [15 ms, 28.6 ms, 42.2 ms], FA = 77°, phase encoding = P‐A (A‐P volumes acquired after each run for unwarping). fMRIPrep 21.0.0 (Esteban et al. 2019) was used to preprocess data. Scanning sessions included a pre‐movie resting‐state scan (8.2 min, 246 TRs), an anxiogenic movie (“Francis”, 5.8 min, 175 TRs), and a post‐movie resting‐state scan (2.3 min, 69 TRs), all using the same scan parameters. The short movie, *Francis* (Hickey 2015), is an animated clip describing the story of a girl who takes a boat out on a lake alone at night. During this boat trip, she repeatedly hears knocking noises on her boat, and tries to escape without success. To validate the anxiogenic nature of the stimulus, we ran a preliminary analysis on phasic skin conductance responses (SCR, per minute), which confirmed physiological arousal (see Supplement 2).

#### 2.1.2 Healthy Brain Network Sample

From the Healthy Brain Network dataset (Alexander et al. 2017), we used motion derivatives of fMRI data (release 9) generated via MRIQC (V 22.0.6; Esteban et al. 2019). Only participants (*n* = 2058) who had available data on age, sex, and parent‐reported anxiety symptoms (SCARED; Birmaher et al. 1997) were analyzed. Participants underwent whole‐brain echo planar imaging on one of three 3T MRIs, using a Siemens Tim Trio or Siemens Prisma, with the following parameters: 2.4 mm^3^ voxels, TR = 800 ms, TE = 3 ms, FA = 31°, phase encoding = A‐P (P‐A volumes acquired after for unwarping), MB = 6. Participants watched a short movie (“The Present”, 3.8 min, 250 TRs), an excerpt from a full‐length movie (“Despicable Me”, 10 min, 750 TRs), and underwent resting‐state scanning (5 min, 375 TRs). For full details of the Healthy Brain Network initiative, see Alexander et al. (2017).

### 2.2 Analysis

We selected three dependent measures: (1) average movement (mean framewise displacement in mm); (2) the percentage of volumes “requiring” censoring; (3) and average global variability in BOLD intensity as a measure of signal quality/noise (DVARS; root mean square of temporal derivatives of whole‐brain signal intensity). The volumes requiring censoring index was based on a commonly used benchmark in fMRI research, framewise displacement > 0.5 mm (Power et al. 2014), with more conservative (> 0.15 mm; Van derwal et al. 2015) and liberal (> 1.00 mm, arbitrary selection) thresholds added as robustness checks for stability of effects. While DVARS and motion are strongly related to one another, including DVARS provided a potentially more direct measure of signal quality (Phạm et al. 2023) and meant analyses were not entirely dependent on realignment parameters. Main analyses were re‐conducted while trimming scan time (i.e., first 65 TRs) to check if scan duration was confounding results. To follow up our primary findings in the NIH dataset, we also investigated average movement (mean framewise displacement in mm) measures in the Healthy Brain Network dataset.

For all group‐level analyses, we used JASP 0.19.1 (Love et al. 2019) and Pingouin (Vallat 2018). For the NIH dataset, we ran mixed ANOVAs including Condition (pre‐movie rest, movie, post‐movie rest) as a within‐subject variable, Group (healthy volunteer, anxiety disorder) a a between‐subjects factor, sex (male, female) as between‐subjects factor, and age as a continuous covariate. Analysis of the Healthy Brain Network dataset was the same except anxiety symptomatology was a continuous covariate. We included two‐way interactions between condition and between‐subject factors/covariates. Continuous covariates were mean‐centered. P‐values were Greenhouse–Geisser corrected when violations of sphericity were apparent for within‐subject contrasts (Mauchly's sphericity tests: *p* < 0.05). To interpret our analyses, we followed up with non‐parametric Wilcoxon tests as data showed non‐normal distributions (i.e., movement floored at 0) and used rank biserial correlations as an effect size metric. Visualizations were generated with Raincloud plots (Allen et al. 2019; kernel density distributions, boxplots, and scatter plots). All analyses used two‐sided tests and *α* = 0.05.

## 3 Results and Summary

### 3.1 Average Movement

**FIGURE 1:**
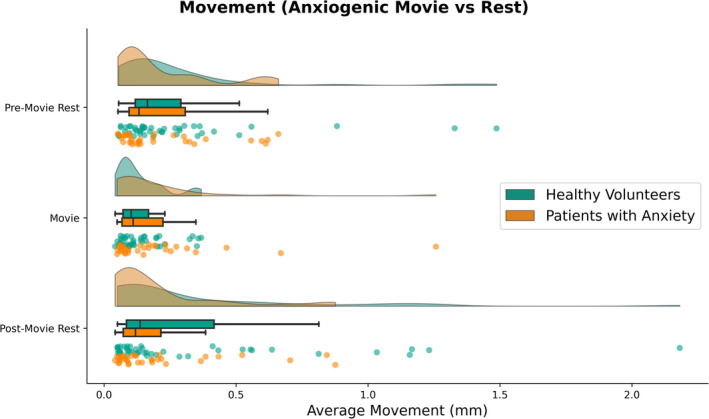
Average movement (mean framewise displacement) across conditions (pre‐movie rest, anxiogenic movie, and post‐movie rest) and diagnostic status (healthy volunteers and patients with anxiety disorders). Kernel density plots for estimated, smoothed distributions; boxplots reflecting median, lower/upper quartiles, and whiskers denoting min/max of data within 1.5 interquartile range; and scatter plots of each data point.

Mixed ANOVA indicated a main effect of Condition on average movement (Table 2; Figure 1). Movement during the anxiogenic movie (*M* = 0.16, SD = 0.17) was significantly lower than during pre‐movie rest (*M* = 0.25, SD = 0.25; *W* = 719, *p* < 0.0001, *r* = −0.53), and post‐movie rest (*M* = 0.28, SD = 0.36; *W* = 965, *p* = 0.004, *r* = −0.37). Pre‐ and post‐movie rest did not significantly differ in average movement (*W* = 1426, *p* = 0.57, *r* = 0.07). Moreover, there was a significant main effect of Age and an Age × Condition interaction. Collapsed across conditions, Age was negatively correlated with average movement (*r* = −0.35, *p* = 0.002), such that younger participants demonstrated higher movement than older participants. The change in motion between conditions was positively correlated with Age (movie—pre‐movie rest: *r* = 0.28, *p* = 0.014; movie—post‐movie rest: *r* = 0.33, *p* = 0.003), such that anxiogenic movie‐watching reduced motion more so for younger participants (who otherwise moved more than older participants). Diagnosis (healthy volunteers, anxiety disorder) and Sex (male, female) were not significantly associated with average movement (*ps* > 0.075).

**TABLE 2:** Mixed ANOVA on average movement.

	d*f*	*F*	*P* (GG‐corr)	*η* ^2^_p_
Within subjects effects				
Condition	1.522	6.179	0.006	0.077
Condition × diagnosis	1.522	2.872	0.075	0.037
Condition × sex	1.522	0.806	0.419	0.011
Condition × age	1.522	6.846	0.004	0.085
Residuals	112.592			
Between subjects effects				
Diagnosis	1	0.51	0.477	0.007
Sex	1	0.141	0.709	0.002
Age	1	7.618	0.007	0.093
Residuals	74			

### 3.2 Censoring

**FIGURE 2:**
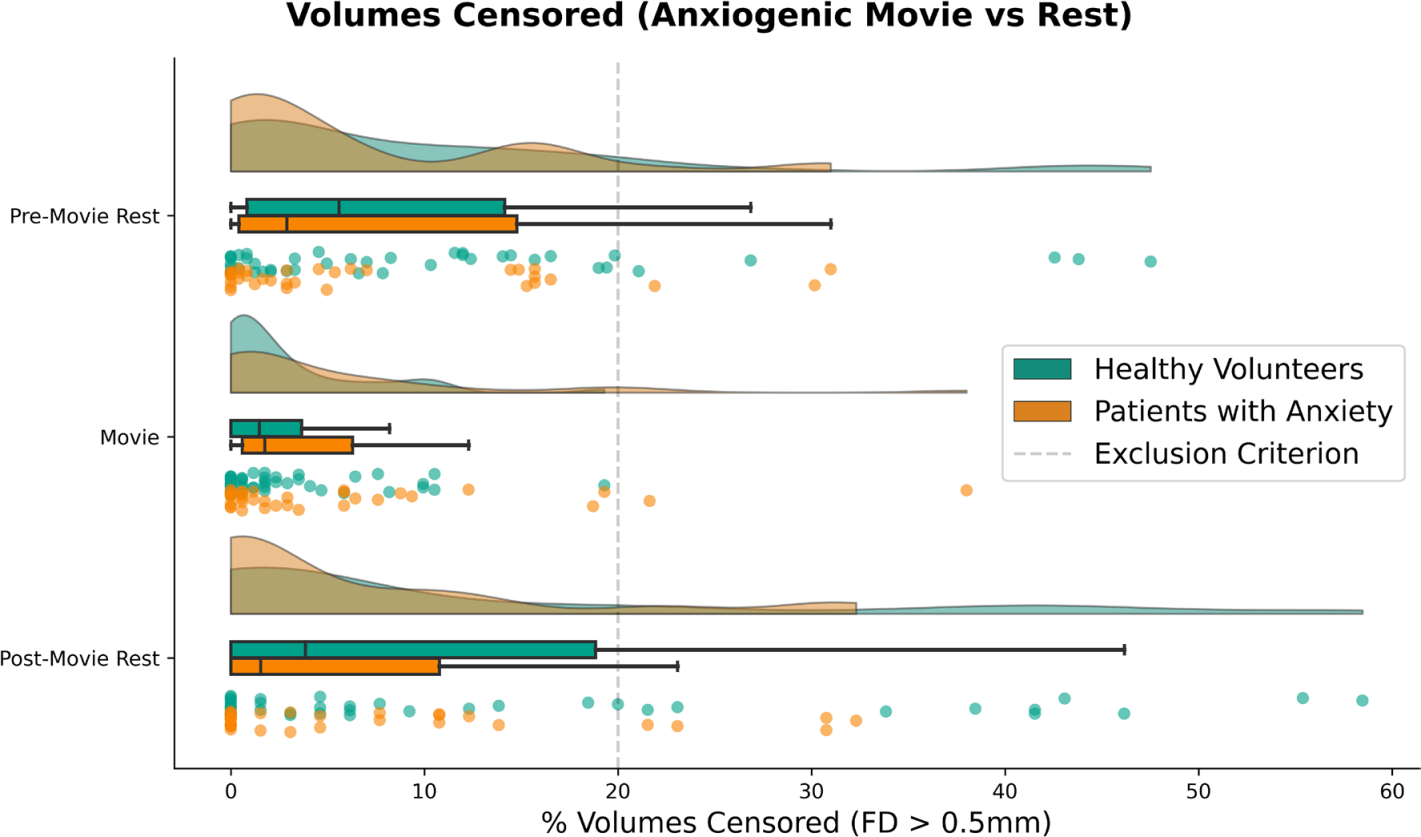
Proportion of volumes censored (framewise displacement [FD] > 0.5 mm) across conditions (pre‐movie rest, anxiogenic movie, and post‐movie rest) and diagnostic status (healthy volunteers and patients with anxiety disorders). Kernel density plots for estimated, smoothed distributions; boxplots reflecting median, lower/upper quartiles, and whiskers denoting min/max of data within 1.5 interquartile range; and scatter plots of each data point.

Mixed ANOVA indicated a main effect of Condition on the percentage of volumes requiring censoring (framewise displacement > 0.5 mm; Table 3; Figure 2). Censoring for the movie (*M* = 4.0, SD = 6.3) was significantly lower than during pre‐movie rest (*M* = 8.5, SD = 10.6; *W* = 650, *p* < 0.0001, *r* = −0.51) and post‐movie rest (*M* = 9.7, SD = 14.5; *W* = 657, *p* = 0.003, *r* = −0.42). Pre‐ and post‐movie rest did not significantly differ in censoring (*W* = 1082, *p* = 0.883, *r* = −0.02). Moreover, there was a main effect of Age and an Age × Condition interaction. Collapsed across conditions, Age was negatively correlated with censoring (*r* = −0.33, *p* = 0.003), such that censoring was higher among younger participants. The change in censoring between conditions was positively correlated with Age (movie—pre‐movie rest: *r* = 0.31, *p* = 0.006; movie—post‐movie rest: *r* = 0.40, *p* < 0.001), such that anxiogenic movie‐watching reduced censoring more so for younger participants (who otherwise required more censoring than older participants).

**TABLE 3:** Mixed ANOVA on censoring.

	d*f*	*F*	*P* (GG‐corr)	*η* ^2^_p_
Within subjects effects				
Condition	1.705	8.800	< 0.001	0.106
Condition × diagnosis	1.705	3.562	0.038	0.046
Condition × sex	1.705	0.532	0.560	0.007
Condition × age	1.705	9.495	< 0.001	0.114
Residuals	126.192			
Between subjects effects				
Diagnosis	1	0.817	0.369	0.011
Sex	1	0.478	0.492	0.006
Age	1	8.995	0.004	0.108
Residuals	74			

There were no main effects of Diagnosis (healthy volunteers, anxiety disorder) or Sex (male, female). There was, however, a Condition × Diagnosis interaction. Healthy volunteers and patients with an anxiety disorder did not significantly differ in the proportion of censored volumes for any one condition (*ps* > = 0.302). However, the overall reduction in censored volumes during the movie appeared significantly greater in healthy volunteers (movie vs. pre‐movie rest: *r* = −0.68, *p* < 0.001; movie vs. post‐movie rest: *r* = −0.62, *p* = 0.001) than in patients (movie vs. pre‐movie rest: *r* = −0.27, *p* = 0.187; movie vs. post‐movie rest: *r* = −0.15, *p* = 0.489), as shown in Figure 2. We note here that the effect size for this interaction with diagnosis (*ηp* ^2^ = 0.046) was under half of those related to age, condition, and age × condition effects (*ηp* ^2^ > 0.1; Table 3).

To contextualize these results in reference to group‐level statistical power, we calculated the number of participants meeting the exclusion criterion based on the commonly used threshold of > 20% volumes censored within a scan (e.g., Michalska et al. 2023). In our sample, 2.6% of participants (*n* = 2) might require exclusion from the movie‐watching analysis. In contrast, 10.3% (*n* = 8) and 19.2% (*n* = 15) of participants might otherwise require exclusion from the pre‐ and post‐movie rest scans, respectively.

### 3.3 Variability in Signal Intensity

**FIGURE 3:**
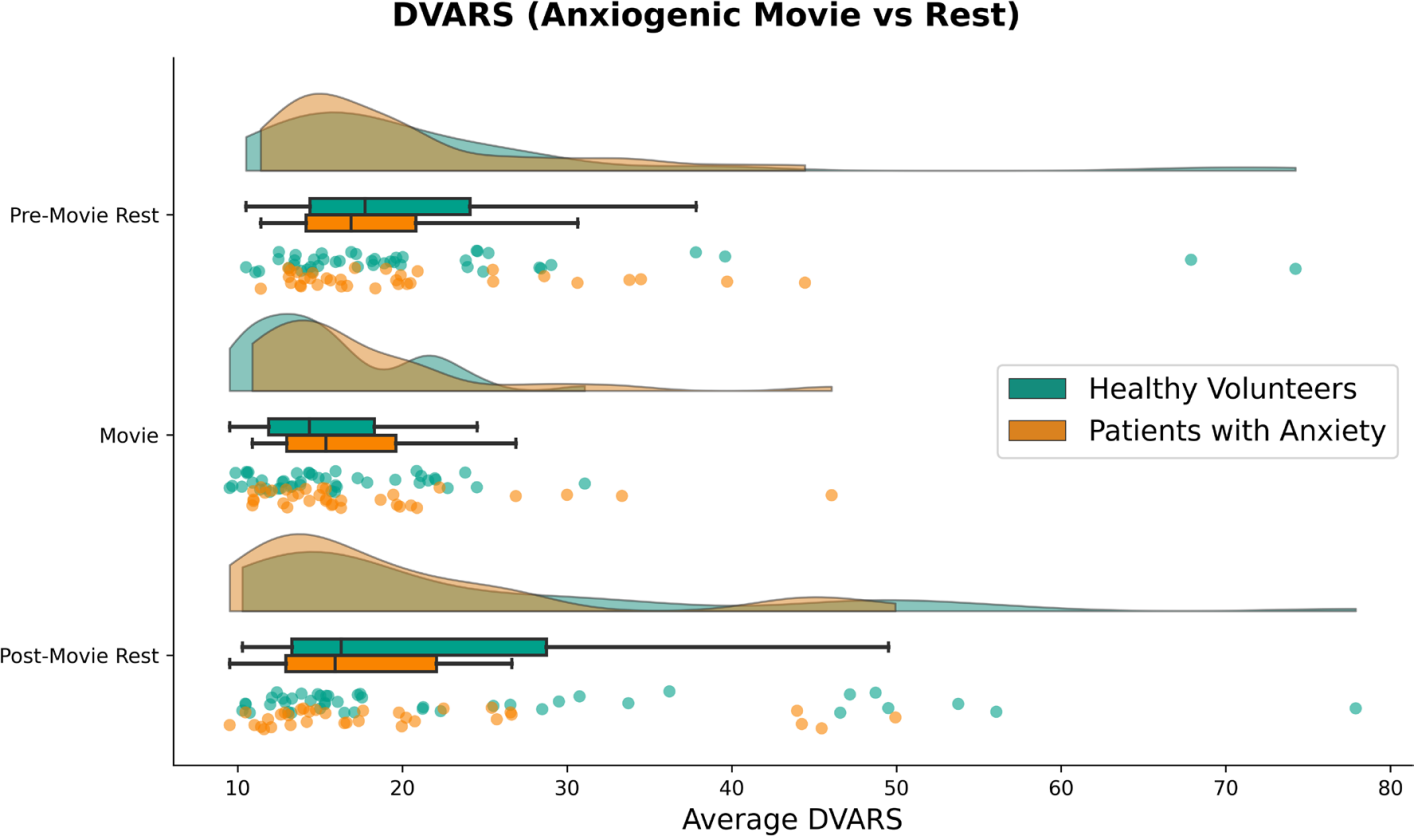
Variability in signal intensity (DVARS) across conditions (pre‐movie rest, anxiogenic movie, and post‐movie rest) and diagnostic status (healthy volunteers and patients with anxiety disorders). Kernel density plots for estimated, smoothed distributions; boxplots reflecting median, lower/upper quartiles, and whiskers denoting min/max of data within 1.5 interquartile range; and scatter plots of each data point.

Mixed ANOVA indicated a main effect of Condition on average DVARS (Table 4; Figure 3). DVARS during the anxiogenic movie (*M* = 16.4, SD = 6.1) was significantly lower than during pre‐movie rest (*M* = 20.7, SD = 10.9; *W* = 732, *p* < 0.001, *r* = −0.52) and post‐movie rest (*M* = 21.9, SD = 13.6; W = 880, *p* = 0.001, *r* = −0.43). Pre‐ and post‐movie rest did not significantly differ in average DVARS (*W* = 1456, *p* = 0.676, *r* = −0.05). Moreover, there was a main effect of Age and an Age × Condition interaction. Collapsed across conditions, Age was negatively correlated with DVARS (*r* = −0.31, *p* = 0.006), such that DVARS was higher among younger participants. The change in DVARS between conditions was positively correlated with Age (movie—pre‐movie rest: *r* = 0.28, *p* = 0.013; movie—post‐movie rest: *r* = 0.31, *p* = 0.007), such that anxiogenic movie‐watching reduced DVARS more so for younger participants (who otherwise had higher DVARS than older participants). Diagnosis (healthy volunteers, anxiety disorder) and Sex (male, female) were not significantly associated with average DVARS (Table 4).

**TABLE 4:** Mixed ANOVA on DVARS.

	d*f*	*F*	*P* (GG‐corr)	*η* ^2^_p_
Within subjects effects				
Condition	1.677	8.990	< 0.001	0.108
Condition × diagnosis	1.677	1.865	0.166	0.025
Condition × sex	1.677	0.101	0.871	0.001
Condition × age	1.677	5.519	0.008	0.069
Residuals	124.071			
Between subjects effects				
Diagnosis	1	0.251	0.618	0.003
Sex	1	0.612	0.437	0.008
Age	1	8.082	0.006	0.098
Residuals	74			

### 3.4 Additional Analyses

#### 3.4.1 Impact of Anxiety Symptoms

Out of the three data quality measures in primary analyses, only one demonstrated a significant interaction with pathological anxiety: anxiogenic movie‐watching reduced censoring in healthy volunteers more so than in patients with anxiety disorders. Given these mixed findings and sample size, a strong inference could not be made regarding the impact of anxiety symptoms on movement during movie‐watching. As such, we conducted a follow‐up analysis in a transdiagnostic sample of participants (*n* = 2058) from the Healthy Brain Network dataset watching non‐anxiogenic movies. Effects of Age, Sex, and Condition were apparent, such that movie‐watching reduced motion, especially amongst younger participants (see Supplement 3). There were no significant main effects of Anxiety (*F*(1, 2054) = 0.156, *p* = 0.693, *ηp* ^2^ < 0.0001) nor a Condition × Anxiety interaction (*F*(1.7, 3450.4) = 0.541, *p* = 0.552, *ηp* ^2^ < 0.001).

#### 3.4.2 Robustness Checks

The main effect of movie‐driven reductions on movement remained stable when using a more conservative censoring threshold (> 0.15 mm; *F*(1.8, 134.2) = 10.14, *p* < 0.001, *ηp* ^2^ = 0.121) and a more liberal threshold (> 1.00 mm; *F*(1.6, 119.9) = 6.10, *p* = 0.005, *ηp* ^2^ = 0.076). The interaction with Diagnosis was not apparent in either of these (*p*s > 0.051). As each condition differed in duration, we re‐calculated key measures using trimmed data for each run (the first 65 volumes only) to match the duration of the condition with the shortest scanning time (post‐movie rest). The main effect of condition was no longer statistically significant for average movement (*F*(1.5, 113.1) = 3.029, *p* = 0.066, *ηp* ^2^ = 0.039), but remained significant for censoring (*F*(1.7, 128.6) = 5.1, *p* = 0.01, *ηp* ^2^ = 0.065) and DVARS (*F*(1.6, 114.8) = 4.391, *p* = 0.022, *ηp* ^2^ = 0.056) measures.

## 4 Summary

Children and adolescents are a difficult‐to‐image population due their tendency to move during scanning sessions (Satterthwaite et al. 2012; Van Dijk et al. 2012). Prior work demonstrates that neutral and positive movie stimuli reduce in‐scanner movement for this population (Frew et al. 2022; Van derwal et al. 2015; Vanderwal et al. 2019). Movies have also started to emerge as a disorder‐relevant stimulus for probing threat‐ and anxiety‐related neural dynamics. Yet, no study has examined how anxiety symptoms and movie‐induced states of anxiety impact in‐scanner motion among youth. One could imagine such anxiogenic media induce excessive movement, given the physiological responding it can elicit. Conversely, the present study demonstrated that anxiogenic movie‐watching was associated with *decreased* in‐scanner motion and increased signal quality compared to resting‐state scanning. There was not sufficient evidence to suggest anxiety symptoms moderate this effect. Even if it does, the effect size of anxiety appears of a far lower magnitude compared to that of age and condition. In sum, our results encourage the use of movie‐watching for studying induced‐ and pathological anxiety on methodological grounds, suggesting movie‐watching may confer potential boosts to statistical power for developmental neuroimaging studies of anxiety via increases to data quality and quantity.

## Conflicts of Interest

The authors declare no conflicts of interest.

## Supporting information


**Data S1.** Supporting Information.

## Data Availability

Code and data for those who consented to share are publicly available on Open Science Foundation (https://osf.io/6v42r/).
