# Supplementary material for: The Relations Among Anxiety, Movie‐Watching, and in‐Scanner Motion
Source: Hum Brain Mapp. 2025 Mar 5;46(4):e70163. doi: 10.1002/hbm.70163 (PMC11880912; doi:10.1002/hbm.70163)
Supplement: Supplementary file 1 — Data S1. Supporting Information. [file HBM-46-e70163-s001.docx]

**Supplementary Materials: The Relations Among Anxiety, Movie-Watching, and In-Scanner Motion**

**Supplement 1**


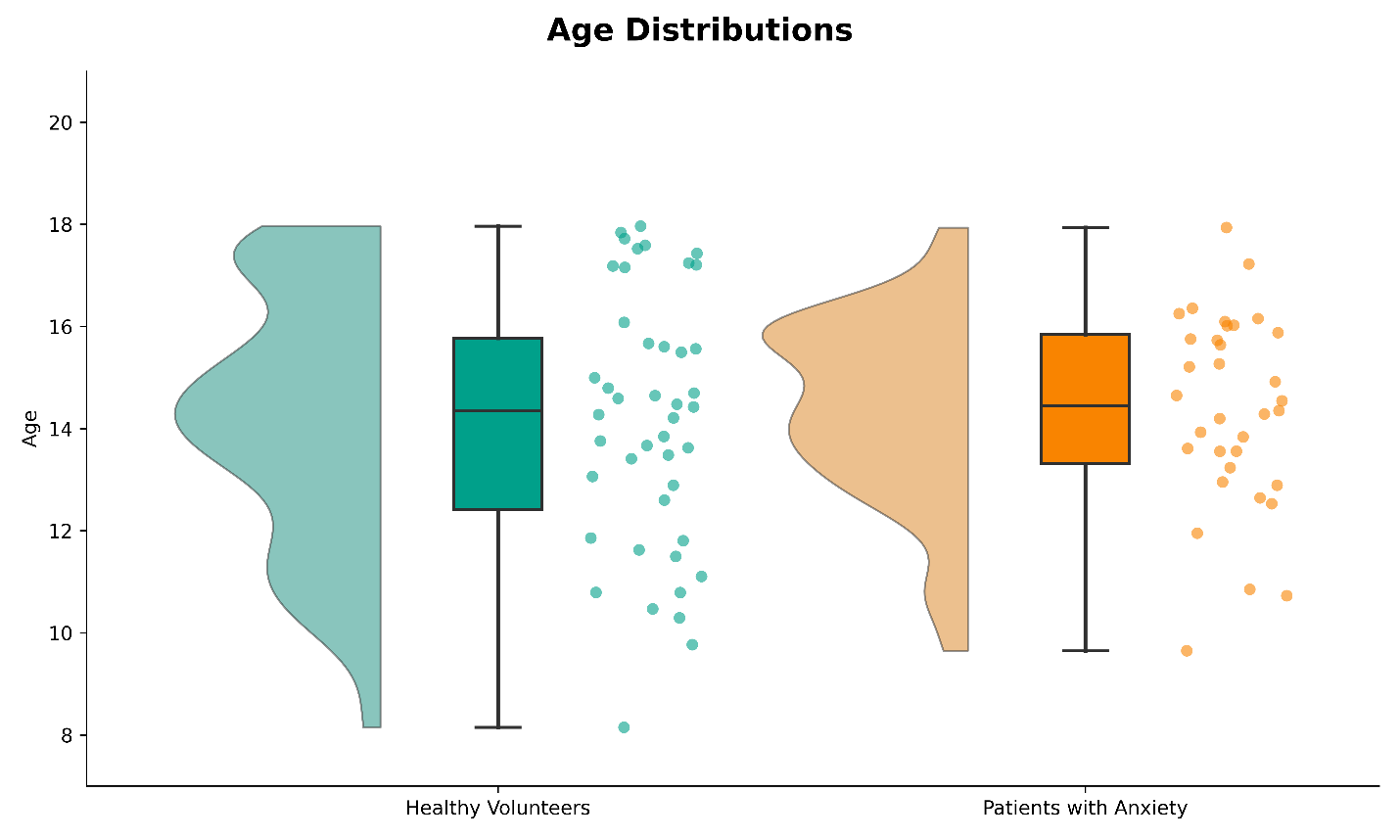
Supplement 2. Variability of age in each group. Kernel density plots for estimated, smoothed distributions; boxplots reflecting median, lower/upper quartiles, and whiskers denoting min/max of data within 1.5 interquartile range; and scatter plots of each data point.

**Supplement 2**

Regarding the NIH sample, physiological arousal during the anxiogenic movie condition was assessed via skin conductance responses (SCR, per minute) from electrodermal signals processed with NeuroKit2 (Makowski et al., 2021). 46 subjects were retained based on biologic limits (0.5 < SCR < 20) and statistical restraints (median absolute deviation; see ‘madmedianrule’ in pingouin, Vallat, 2018). Mixed ANOVA indicated main effects of Condition, but not main effects of Sex, Diagnosis, or Age (**Supplement 2 Table**), on SCR. SCR was significantly higher during movie-watching (M=2.89, SD=1.74) compared to pre-movie rest (M=2.00, SD=1.14; W=267, *p*=.003) but not post-movie rest (M=2.51, SD=1.48; W=599, *p*=.526). Pre- and post-movie rest did not significantly differ in average SCR (W=699, *p*=.084). This main effect was qualified by a significant interaction. The increase in SCR between rest and movie-watching was greater in patients (movie vs. pre-movie rest: W=39, *p*=.006; movie vs. post-movie rest: W=53, *p*=.029) than in healthy volunteers (movie vs. pre-movie rest: W=153, *p* = .105; movie vs. post-movie rest: W=259, *p* = .368).

| **Supplement 2 Table**. Mixed ANOVA on SCR | | | | |
| --- | --- | --- | --- | --- |
|  | df | F | P | η²_p_ |
| *Within Subjects Effects* | | | | |
| Condition | 2 | 7.533 | < .001 | 0.152 |
| Condition X Diagnosis | 2 | 3.418 | 0.037 | 0.075 |
| Condition X Sex | 2 | 2.308 | 0.106 | 0.052 |
| Condition X Age | 2 | 0.608 | 0.547 | 0.014 |
| Residuals | 84 |  |  |  |
| *Between Subjects Effects* | | | | |
| Diagnosis | 1 | 1.139 | 0.292 | 0.026 |
| Sex | 1 | 1.753 | 0.193 | 0.040 |
| Age | 1 | 0.156 | 0.695 | 0.004 |
| Residuals | 42 |  |  |  |

Makowski, D., Pham, T., Lau, Z. J., Brammer, J. C., Lespinasse, F., Pham, H., Schölzel, C., & Chen, S. H. A. (2021). NeuroKit2: A Python toolbox for neurophysiological signal processing. *Behavior Research Methods*, *53*(4), 1689–1696. <https://doi.org/10.3758/s13428-020-01516-y>

**Supplement 3**

We conducted a follow-up analysis in a transdiagnostic sample participants (n=2058) from the Healthy Brain Network dataset watching non-anxiogenic movies. Effects of Age, Sex, and Condition were apparent, such that movie-watching reduced motion, especially amongst younger participants. No significant main effects nor interactions with anxiety symptoms emerged. Mixed ANOVA results for Healthy Brain Network data (**Supplement 3 Table**). Age was negatively associated with average movement in all conditions (rest and movies; *rs* < -.17, *ps* < .001). However, the within-subject change in average movement between conditions was significantly associated with age (Rest – Despicable Me: *r* = -.11, *p* < .001; Rest – The Present: *r* = -.11, *p* < .001), replicating prior effects that movie-attenuated motion is greatest amongst younger participants. There were no effects related to anxiety.

| **Supplement 3 Table**. Mixed ANOVA on Average Movement (Healthy Brain Network) | | | | |
| --- | --- | --- | --- | --- |
|  | df | F | P | η²_p_ |
| *Within Subjects Effects* | | | | |
| Condition | 1.680 | 21.602 | < .001 | 0.010 |
| Condition X Anxiety | 1.680 | 0.541 | 0.552 | <.001 |
| Condition X Sex | 1.680 | 1.316 | 0.266 | < .001 |
| Condition X Age | 1.680 | 18.655 | < .001 | 0.009 |
| Residuals | 3450.372 |  |  |  |
| *Between Subjects Effects* | | | | |
| Anxiety | 1 | 0.156 | 0.693 | <.0001 |
| Sex | 1 | 30.568 | < .001 | 0.015 |
| Age | 1 | 192.036 | < .001 | 0.085 |
| Residuals | 2054 |  |  |  |
